# Prevalence of Malnutrition and Associated Factors of Stunting among 6–23-Month-Old Infants in Central Rural China in 2019

**DOI:** 10.3390/ijerph18158165

**Published:** 2021-08-02

**Authors:** Jing Liu, Jing Sun, Jian Huang, Junsheng Huo

**Affiliations:** Key Laboratory of Trace Element Nutrition of National Health Commission (NHC), National Institute for Nutri-tion and Health, Chinese Center for Disease Control and Prevention, No. 27 Nanwei Road, Xicheng District, Beijing 100050, China; liujingjenny1989@163.com (J.L.); sunjing@ninh.chinacdc.cn (J.S.); huangjian@ninh.chinacdc.cn (J.H.)

**Keywords:** malnutrition, wasting, stunting, underweight, 6–23-month-old infants, cross-sectional survey

## Abstract

This study aimed to evaluate the prevalence of malnutrition and to investigate the associated factors of stunting among 6–23-month-old infants in poor rural areas of central China. The China Nutrition Improvement Project on Children in Poor Areas was conducted in 56 national-level poor counties of seven provinces in 2019. We performed a multivariate binary logistic regression analysis to determine the associated factors of stunting. This cross-sectional study included 17,193 infants. The overall prevalence of stunting was the highest (3.9%), followed by overweight (3.0%), underweight (2.1%), wasting (2.0%), and obesity (0.5%). Girls [OR = 0.55, 95% CI (0.46, 0.65)], infants meeting requirements of minimum diversity diet [OR = 0.81, 95% CI (0.67, 0.98)], and mothers with middle-school or high-school education and above decreased the prevalence of stunting. Infants with diarrhea in two weeks [OR = 1.26,95% CI (0.98, 1.62)] were at higher odds of stunting. The malnutrition status in the study areas was improved, and vulnerable infants were found to need additional and earlier monitoring to detect and fundamentally prevent undernutrition.

## 1. Introduction

Malnutrition has been defined as a pathological state, including undernutrition and overnutrition [1]. Infants are the most vulnerable population when it comes to malnutrition. Globally, 144.0 million, 47.0 million, and 38 million children under 5 years old suffered from stunting, wasting, and overweight in 2019, respectively [2]. In China, the overall prevalence of stunting, underweight, and wasting of 0–5-year-old children was 8.1%, 2.4%, and 1.9%, respectively, in 2013, which represented a decline compared to the prevalence of the same in 2002 and a more considerable reduction in rural areas than in urban areas [3]. In rural areas, the prevalence of malnutrition was higher than that in the whole of China. The total prevalence of malnutrition, stunting, and overweight was 19.2%, 8.4%, and 8.8%, respectively, among 0–5-year-old infants and children in China’s poor rural areas in 2016 [4].

Around the world, malnutrition of infants and children continues to be the leading public health problem in low- and middle-income countries [4,5,6]. Undernutrition of infants has been associated with lower human capital, high glucose concentrations, blood pressure, and harmful lipid profiles [7]. Based on a study in Lancet, stunting was found to be a key risk factor that requires urgent intervention [8]. Height-for-age (HAZ) for 0–2-year-olds infants was the best predictor of human capital, and stunting of infants was associated with a higher rate of overweight and chronic diseases, including diabetes, cardiovascular disease, breast and other cancers, and mental health issues in later life [9,10]. Some studies have reported that early childhood stunting is related to greater central adiposity for adults, including triceps skinfold and waist-to-hip ratio after adjusting for overall fatness and confounders [11,12]. In the last few decades, there has been significant progress in improving nutritional status in poor rural areas. However, there has been no timely report focus on the prevalence of malnutrition in poor rural areas.

Previous studies described the malnutrition status of a large sample size among children under five years old throughout China. However, there are few data regarding the prevalence of malnutrition among 6–23-month-old infants in poor rural areas. Our study firstly used the Child Growth Standard of the WHO to assess the prevalence of malnutrition among 6–23-month-old infants in 2019 [13]. We used a large sample from poor rural areas in central China (PRC). The primary aim was to determine the current prevalence of malnutrition among 6–23-month-old infants in PRC. Additionally, due to the high prevalence of stunting, we also investigated its associated factors.

## 2. Materials and Methods

### 2.1. Study Population

Our data were from the Nutrition Improvement Project on Children in Poor Areas of China (NIPCPAC) in 2019. The NIPCPAC is a nationally representative survey that used multiple sampling methods to select eligible 6–23-month-old infants in poor rural areas of China. The multiple sampling methods included province stratification, multi-stage sampling, PPS sampling, and systematic random sampling. A total of 45,221 infants were sampled out of 19 provinces, and 6–23-month-old infants were selected. Our research included seven provinces, including Hunan, Hubei, Henan, Hebei, Anhui, Jiangxi, and Shanxi. There were 18,022 participants in our research, and data for 17,193 participants were analyzed after data cleaning.

The study was approved by the Ethical Committee of the National Institute for Nutrition and Health in the Chinese Center for Disease Control and Prevention, ethical reference number 2014-001. All caregivers of infants provided written informed consent.

### 2.2. Data Collection

#### 2.2.1. Questionnaires

We used standard questionnaires designed by the experts of NIPCPAC. The questionnaires were used to collect data regarding the essential characteristics of infants, including sex and age. We also gathered information regarding their parents or caregivers, including the level of education, ethnicity, and occupation. We also collected information regarding feeding patterns and the status of 24-h complementary food intake based on the WHO [14].

#### 2.2.2. Anthropometry

In the study, we used a body weight and height scale, which can display the results of infants and children digitally. All weight and height scales selected by surveillance counties must meet the national quality standards [15]. Body length was measured and recorded with an accuracy of 0.1 cm. Body weight was measured in kilograms with an accuracy of 0.05 kg.

### 2.3. Study Variables

We calculated the Z score using an Anthro Survey Analyzer and evaluated the nutritional status of infants according to the WHO Child Growth Standard 2006. We used three indicators: length for age (HAZ), weight for age (WAZ), and weight for height (WHZ). A HAZ score <−2 was defined as stunting; a WAZ score <−2 was defined as underweight; a WHZ score <−2 was defined as wasting. Overweight was defined as a WHZ score >2. Any one or more of the above four conditions defined malnutrition. Malnutrition included undernutrition and overnutrition.

### 2.4. Statistical Analysis

For analysis, the age of infants was categorized as 6–11 months (6–11 mon), 12–17 months (12–17 mon), and 18–23 months (18–23 mon). We divided primary caregivers into groups based on whether they were parents or grandparents. Education was categorized into three groups, i.e., those who had graduated from primary school or below (primary/below), middle school, and high school or above (high school/above). The ethnicity of parents was divided into Han and minorities. We divided birthweight into three groups: <2500 g was the low birth weight (LBW), 2500~4000 g was the normal birth weight (NBW), and ≥4000 g was macrosomia (MW). We calculated the minimum diversity diet (MDD) based on the WHO standard [16,17]. Occupation was divided into four groups: no work, work related to agriculture, work related to the service sector, and other types of work.

We used the Pearson X^2^ test to compare binary and categorical variables. A multiple logistic regression analysis was conducted to assess associated factors of stunting with a forwarding likelihood ratio. To avoid collinearity, before the regression, we used the collinearity test. In our study, the tolerance is much larger than 0.1, and the variance inflation factor is less than 10, so there is no collinearity. The logistic regression model included all potential influencing factors identified based on the chi-square test. All statistical tests were 2-sided, and the significance level was set at less than 0.05. We evaluated the associated factors of stunting via multiple logistic regression. The odds ratio (OR) and 95% confidence intervals (CIs) were calculated. We carried out statistical analysis using IBM SPSS Statistics for Windows, version 19 (IBM Corp., Armonk, NY, USA).

## 3. Results

### 3.1. Characteristics of Surveyed Children and Their Caregivers

Table 1 shows the characteristics of surveyed children and their caregivers. A total of 17,193 6–23-month-old infants were enrolled. There were 8927 (51.9%) boys and 8266 (48.1%) girls. The number of people was roughly equal in each month group. Approximately 3.5% of infants were categorized as low birth weight, 5.4% were macrosomia, and 4.4% were preterm births. More than 80% of infants had not experienced fever or diarrhea in the last two weeks. Roughly 92% of parents were of Han nationality, and around 8% of parents were minorities. More than half of the parents had graduated from middle school, and about 7.1% of mothers and 4.8% of fathers had primary education and below. Over half of the mothers were homemakers, and 7.7% of mothers had jobs related to agriculture. Approximately 90% of fathers were in work, and 74.4% of fathers were employed with other jobs, such as in the service and manufacturing industries; only 15.4% of fathers had jobs related to agriculture.

### 3.2. Nutritional Status of 6–23-Month-Old Infants

Table 2 describes the mean and SD of nutritional status in the different age groups. The overall mean body weight and length were 10.3 kg and 77.8 cm, and means for boys were significantly higher than means for girls (*p* < 0.05). The mean of body length and weight increased significantly with age. The overall mean of HAZ was highest in the 6–11-month-old infants, and it decreased significantly with age (*p* < 0.05). The overall mean of HAZ in boys was significantly lower than in girls, and the trend was the same in each age group. The means of WAZ, WHZ, and BMIZ decreased significantly with age, and the highest means occurred in the 6–11-month-old age group.

### 3.3. Prevalence of Malnutrition in 6–23-Month-Old Infants

Table 3 presents the prevalence of malnutrition in 6–23-month-old infants based on the factors of infants and parents. Amongst all infants, the prevalence of stunting was the highest (3.9%), followed by overweight (3.0%), underweight (2.1%), wasting (2.0%), and obesity (0.5%). The prevalence of stunting and underweight increased with age, while overweight and fat had the opposite tendency (*p* < 0.05). The prevalence of wasting fluctuated, having been found to be 2.2%, 1.9%, and 2.0% in the 6–11-month-old, 12–17-month-old, and 18–23-month-old groups, respectively. The prevalence of overweight and obesity was the highest in 6–11-month-old infants and decreased with age. The prevalence of malnutrition was much higher among boys than among girls, and each age group had the same trend (*p* < 0.05).

### 3.4. Associated Factors of Stunting in 6–23-Month-Old Infants

Table 4 shows the multivariate logistic analysis results associated with stunting among 6–23-month-old infants in poor rural areas. Girls (OR = 0.55, 95% CI (0.46, 0.65)) were associated with a lower prevalence of stunting than boys. The prevalence of stunting increased with age (OR = 1.05, 95% CI (1.03, 1.07)). Among infants who met the requirements for minimum diversity diet (MDD), the prevalence of stunting decreased (OR = 0.81, 95% CI (0.67, 0.98)). Infants whose mothers were minorities had a lower prevalence of stunting than those with a mother of Han nationality (OR = 0.63, 95% CI (0.44, 0.90)). Mothers with middle school (OR = 0.67, 95% CI (0.51, 0.88)) and high school or above (OR = 0.49, 95% CI (0.37, 0.65)) education were less likely to be stunted. Mothers working in agriculture (OR = 1.42, 95% CI (1.07, 1.89)) were at lower odds of stunting than homemakers. Paternal occupation and education were not significantly associated with the prevalence of stunting.

## 4. Discussion

This study indicated the prevalence of malnutrition among 6–23-month-old infants in poor rural areas of central China in 2019. We evaluated the prevalence of malnutrition based on the WHO indicators of Child Growth Standards. There were 17,193 6–23-month-old infants, and the prevalence of stunting was the highest (3.9%), followed by overweight (3.0%), underweight (2.1%), wasting (2.0%), and obesity (0.5%). This was much better than the prevalence of stunting (21.9%), wasting (7.3%), and overweight (5.9%) among children under five years of age all over the world in 2018 [17]. More than half of the world’s stunted children were in Asia [18]. However, the rate of stunting in China was much better than that in other Asian countries among infants aged 0–23 months, such as India (33%) and the Maldives (20%) [19]. We investigated the associated factors of stunting in parents and infants via the multiple logistic regression model. The results showed that the prevalence of stunting was associated with month, gender, complementary feeding, maternal ethnicity, maternal occupation, and maternal educational level.

Multiple factors determine child growth. Quality complementary feeding is undoubtedly one of these determinants. In our study, the qualified MDD requirement is a prospective factor of stunting. Infants older than six months need to have breastmilk and are introduced complementary meals in time [20,21]. Globally, malnutrition of children is primarily caused by inadequate feeding practices among infants and young children [2]. In many poor areas of China, inadequate MDD was positively associated with a higher stunting rate among children aged 3 years old [22]. Based on 11 demographic and health surveys, dietary diversity was significantly associated with HAZ and stunting [23]. Previous studies have illustrated that a lack of dietary diversity will contribute to the malnutrition status of infants, especially stunting [23,24,25]. Unfortunately, studies have shown that inappropriate feeding practices for 6–23-month-old infants are common in many rural areas of China [26]. To improve the malnutrition status of 6–23-month-old infants, the government and relevant departments should promote breastfeeding and scale up complementary feeding education in poor rural areas.

The prevalence of stunting was found to be associated with the monthly age of infants. Childhood undernutrition worsened with the increasing age of infants. The prevalence of stunting increased with month, consistent with research results both in China and abroad [3,22,27,28]. Our findings revealed that infants with mothers with higher education levels were less likely to be stunted than those with mothers with primary school education or below. In rural western China, the mother’s education level was negatively associated with childhood undernutrition [27]. A parent with a lower education level may have a low salary and little knowledge of feeding practices. Due to insufficient food supplies and scientific feeding knowledge, parents may only introduce grain foods as complementary food and breastmilk to infants [22]. However, in a study in Bangladesh, the authors found that higher education among mothers and better household socioeconomic conditions were not sufficient to decrease malnutrition rates among infants [29]. In our study, infants of minority mothers had a lower prevalence of stunting than those with Han parents, which was inconsistent with the findings of past studies [4,27]. In previous research, ethnic minorities, including Tibetan, Uighur, and Yi, were associated with a higher prevalence of stunting due to inconvenient traffic infrastructure, low income, and limited food resources [30].

Malnutrition includes undernutrition and overnutrition, such as overweight and obesity [1]. In our study, the prevalence of overweight and obesity decreased compared with 10% of overweight among 2–6-year-old children in the 2009–2011 China Health and Nutrition Survey [31]. Overweight and obese infants are becoming an increasingly severe contributor to adult obesity, diabetes, and chronic diseases [8]. Few studies have recognized the problem of double burden in malnutrition in impoverished areas in China. In low-income groups, overweight and obesity prevalence was 13.5% and 9.9%, respectively, among 2–6-year-old children in 2011 [31]. In children under five, the prevalence of overweight was 5.4% in a poor midwestern China area in 2009, which represents a newly emerging problem [31]. The prevalence of overweight was found to be higher in urban and high-income areas in past research [31]. Given that child malnutrition in poor rural areas is often ignored, we should provide nutritional education including guidance around controlling undernutrition and overnutrition to parents at the same time.

A strength of our study was that we first used the data of 6–23-month-old infants to assess malnutrition in poor rural areas in central China in 2019. Additionally, our data came from the national program, and so, they were of good quality and high reliability. One potential limitation is that the causal relationships between sociodemographic characteristics and stunting need more analysis to confirm the cross-sectional design of this study. In addition, family income and the weight and height of mothers were not assessed in our questionnaire. Because the prevalence of malnutrition was different in each month group, we would analyze the associated factors of stunting in 6–11 month, 12–17 month and 18–23 month infants in the future, respectively.

## 5. Conclusions

In conclusion, our study showed the prevalence of stunting; wasting; and underweight, overweight, and obesity among 6–23-month-old infants in poor areas of central China in 2019. Malnutrition is a complex problem affected by a range of determinants. Infant age, male gender, inadequate MDD, and lower education level of mothers were risk factors for stunting. Specifically, infants in rural areas of China need additional and earlier monitoring to detect and prevent undernutrition, fundamentally reducing the overall burden of undernutrition. Follow-up studies may comprehensively consider nutritional status in the first 1000 days of life, such as collecting more information on pregnancy and the status of mothers.

## Figures and Tables

**Table 1:** Characteristics of surveyed children and their caregivers (*n* = 17,193).

Characteristic (*n*, %)		Sample *(n,* %)
IYC		
Months		
	6–11 months	5679 (33.0)
	12–17 months	5689 (33.1)
	18–23 months	5825 (33.9)
Sex		
	Boy	8927 (51.9)
	Girl	8266 (48.1)
Preterm birth		764 (4.4)
Birth weight		
	<2500 g	607 (3.5)
	2500 g~4000 g	15,653 (91.0)
	≥4000 g	933 (5.4)
Two-week morbidity		
	Fever	1740 (10.1)
	Diarrhea	1719 (10.0)
Mother		
Ethnicity		
	Han	15,924 (92.6)
Education		
	Primary/below	1218 (7.1)
	Middle school	9702 (56.5)
	High school/above	6246 (36.4)
Occupation		
	No work	9531 (55.4)
	Agriculture	1317 (7.7)
	other work	6345 (36.9)
Father		
Ethnicity		
	Han	15,892 (92.4)
Education		
	Primary/below	830 (4.8)
	Middle school	9705 (56.4)
	High school/above	6623 (38.5)
Occupation		
	No work	1766 (10.3)
	Agriculture	2644 (15.4)
	other work	12,783 (74.4)

**Table 2:** Nutritional status of 6–23-month-old infants.

Characteristic	Gender	Month Group (Mean ± SD)			Total	*p*-Value
		6–11 Months	12–17 Months	18–23 Months		
Body weight (kg) ^1^	Boy	9.4 ± 1.2 *	10.6 ± 1.2 *	11.7 ± 1.3 *	10.6 ± 1.5 *	<0.001
	Girl	8.8 ± 1.1	10.0 ± 1.2	11.1 ± 1.3	10.0 ± 1.5	<0.001
	Total	9.1 ± 1.2	10.3 ± 1.2	11.4 ± 1.3	10.3 ± 1.6	<0.001
Body length (cm)	Boy	72.2 ± 3.3 *	78.5 ± 3.5 *	84.1 ± 3.6 *	78.3 ± 6.0 *	<0.001
	Girl	70.9 ± 3.3	77.4 ± 3.5	83.1 ± 3.6	77.2 ± 6.1	<0.001
	Total	71.6 ± 3.4	78.0 ± 3.5	83.6 ± 3.6	77.8 ± 6.1	<0.001
HAZ	Boy	−0.02 ± 1.16 *	−0.17 ± 1.15 *	−0.32 ± 1.14 *	−0.17 ± 1.16 *	<0.001
	Girl	0.13 ± 1.11	0.00 ± 1.05	−0.18 ± 1.03	−0.02 ± 1.07	<0.001
	Total	0.06 ± 1.14	−0.09 ± 1.11	−0.25 ± 1.09	−0.10 ± 1.12	<0.001
WAZ	Boy	0.14 ± 1.08	−0.05 ± 1.02 *	−0.16 ± 0.98 *	−0.02 ± 1.04 *	<0.001
	Girl	0.18 ± 0.98	0.05 ± 0.92	−0.08 ± 0.90	0.05 ± 0.94	<0.001
	Total	0.16 ± 1.03	−0.01 ± 0.98	−0.12 ± 0.94	0.01 ± 0.99	<0.001
WHZ	Boy	0.25 ± 1.14	0.04 ± 1.04	−0.00 ± 0.99	0.10 ± 1.06	<0.001
	Girl	0.21 ± 1.04	0.06 ± 0.98	−0.03 ± 0.94	0.08 ± 0.99	<0.001
	Total	0.23 ± 1.09	0.05 ± 1.01	−0.02 ± 0.97	0.09 ± 1.03	<0.001
BMIZ	Boy	0.20 ± 1.16 *	0.06 ± 1.06	0.06 ± 1.02	0.11 ± 1.08	<0.001
	Girl	0.14 ± 1.06	0.07 ± 0.99	0.04 ± 0.97	0.08 ± 1.01	<0.001
	Total	0.17 ± 1.11	0.07 ± 1.03	0.05 ± 1.00	0.10 ± 1.05	<0.001

^1^ The *t*-test was used to compare the mean values of boys and girls, and *p* < 0.05 was marked as * located in the data of boys.

**Table 3:** Prevalence of malnutrition among 6–23-month-old infants.

Malnutrition Prevalence		Month Group (*n*, %)			Total	*p*-Value
		6–11 Month	12–17 Month	18–23 Month		
Stunting	Male	108 (3.6)	145 (4.9)	193 (6.4)	446 (5.0)	<0.001
	Female	57 (2.1)	71 (2.6)	101 (3.6)	229 (2.8)	<0.001
	Total	165 (2.9)	216 (3.8)	294 (5.0)	675 (3.9)	<0.001
Underweight	Male	71 (2.4)	86 (2.9)	80 (2.7)	237 (2.7)	0.454
	Female	44 (1.6)	33 (1.2)	51 (1.8)	128 (1.5)	0.185
	Total	115 (2.0)	119 (2.1)	131 (2.2)	365 (2.1)	0.693
Wasting	Male	83 (2.8)	67 (2.3)	57 (1.9)	207 (2.3)	0.074
	Female	44 (1.6)	42 (1.5)	57 (2.0)	143 (1.7)	0.351
	Total	127 (2.2)	109 (1.9)	114 (2.0)	350 (2.0)	0.42
Overweight	Male	139 (4.7)	85 (2.9)	72 (2.4)	296 (3.3)	<0.001
	Female	102 (3.8)	67 (2.5)	46 (1.6)	215 (2.6)	<0.001
	Total	241 (4.2)	152 (2.7)	118 (2.0)	511 (3.0)	<0.001
Obesity	Male	30 (1.0)	11 (0.4)	8 (0.3)	49 (0.5)	<0.001
	Female	21 (0.8)	8 (0.3)	5 (0.2)	34 (0.4)	<0.001
	Total	51 (0.9)	19 (0.3)	13 (0.2)	83 (0.5)	<0.001

**Table 4:** Multivariate logistic analysis ^2^ of stunting.

Variables		β	SE	OR (95% CI)	*p*-Value
Month		0.05	0.01	1.05(1.03, 1.07)	<0.001
Gender		−0.61	0.09	0.55(0.46, 0.65)	<0.001
Mother Education					<0.001
	Middle school vs. primary/below	−0.4	0.14	0.67(0.51, 0.88)	<0.001
	High/above vs. primary/below	−0.72	0.15	0.49(0.37, 0.65)	<0.001
Mother Occupation					0.07
	Agriculture vs. Unemployed	0.35	0.14	1.42(1.07, 1.89)	0.01
	Business vs. Unemployed	−0.06	0.16	0.94(0.69, 1.28)	0.71
	Others work vs. Unemployed	−0.03	0.1	0.97(0.80, 1.18)	0.77
Mother minority		−0.47	0.18	0.63(0.44, 0.90)	0.01
MDD ^1^ qualified		−0.21	0.1	0.81(0.67, 0.98)	0.03
Constant		−2.16	0.29	0.12	<0.001

^1^ MDD: minimum acceptable diet. ^2^ The model was controlled for birth weight and sickness in the last two weeks.

## Data Availability

The datasets presented in this article are not readily available because of the regulations of Data Management in the Chinese Center for Disease Control and Prevention. Requests to access the datasets should be directed to the National Institute for Nutrition and Health, Chinese Center for Disease Control and Prevention, the email address of which is chinanutrition@chinacdc.cn.
